# Assessing Associations between Socio-Economic Environment and Self-Reported Health in Amsterdam Using Bespoke Environments

**DOI:** 10.1371/journal.pone.0068790

**Published:** 2013-07-17

**Authors:** Eleonore M. Veldhuizen, Karien Stronks, Anton E. Kunst

**Affiliations:** 1 Department of Human Geography, Planning & International Development Studies, Faculty of Social and Behavioural Sciences, University of Amsterdam, Amsterdam, The Netherlands; 2 Department of Public Health, Academic Medical Center, University of Amsterdam, Amsterdam, The Netherlands; Old Dominion University, United States of America

## Abstract

**Background:**

The study of the relationship between residential environment and health at micro area level has a long time been hampered by a lack of micro-scale data. Nowadays data is registered at a much more detailed scale. In combination with Geographic Information System (GIS)-techniques this creates opportunities to look at the relationship at different scales, including very local ones. The study illustrates the use of a ‘bespoke environment’ approach to assess the relationship between health and socio-economic environment.

**Methods:**

We created these environments by buffer-operations and used micro-scale data on 6-digit postcode level to describe these individually tailored areas around survey respondents in an accurate way. To capture the full extent of area effects we maximized variation in socio-economic characteristics between areas. The area effect was assessed using logistic regression analysis.

**Results:**

Although the contribution of the socio-economic environment in the explanation of health was not strong it tended to be stronger at a very local level. A positive association was observed only when these factors were measured in buffers smaller than 200 meters. Stronger associations were observed when restricting the analysis to socioeconomically homogeneous buffers. Scale effects proved to be highly important but potential boundary effects seemed not to play an important role. Administrative areas and buffers of comparable sizes came up with comparable area effects.

**Conclusions:**

This study shows that socio-economic area effects reveal only on a very micro-scale. It underlines the importance of the availability of micro-scale data. Through scaling, bespoke environments add a new dimension to study environment and health.

## Introduction

Since the mid-1990s, a great deal of research has been conducted with the aim to assess area effects on health (for an overview, see Smyth) [1]. A key aim in this research has been to demonstrate the independent effect, if any, that area-level socio-economic factors have on health. Most studies have concluded that living in a socio-economically disadvantaged neighbourhood is associated with only relatively small effects on health outcomes. Furthermore, the health effects shown in observational studies often disappear after extensive adjustment for individual socio-economic characteristics (see for example, Robert, Pickett et al., Reijneveld, Yen et al.) [2]–[5].

An explanation for this lack of strong association may be that area effects are difficult to measure. It is widely recognized that the selection of the spatial unit is an important consideration in accurately detecting area effects. However, the definitions of ‘neighbourhood’ used in most studies are not based on theoretical considerations but instead on data availability [6]. As a result, in many cases, administratively defined areas have been used to define the spatial units for analysis.

Using administratively defined areas poses two types of problems related to, respectively, scale effects and boundary effects. **Scale effects** refer to the influence of the spatial scale used on the measurement of area effects. It is commonly agreed upon that the existence and strength of area effects on health are scale dependent [7]–[12]. Generally, stronger effects may be found if a smaller spatial scale is used [13]. **Boundary effects** occur especially when administrative boundaries do not accurately reflect appropriate neighbourhood boundaries. Administrative borders may not be relevant in the daily lives of residents. Residents living near the border of administrative areas may relate more to neighbouring administrative zones [14]. Due to such effects, the use of administratively defined areas may underestimate or skew geographical effects that would otherwise be observed within more relevantly defined areas.

Scale and boundary effects could in principle be avoided when so-called “bespoke environments” are used. In this approach, separate neighbourhoods are created for each individual resident. These neighbourhoods are centered on each respondent’s home and are independent of administrative boundaries. The size of these neighbourhoods can be determined flexibly in terms of distance (the radius of the buffer) or counts (e.g. the number of residents).

This methodology has been applied in several research fields. Bespoke environments were introduced in the 1990s in studies of voting [15] and of social exclusion [16]. Studies on voting behaviour observed clear links between the characteristics of local milieus and voting behaviour [15], [17]. Anderson et al. [11] used, aside from administrative units, bespoke environments of 100 meters around each individual’s home to study area effects on income. They found area effects to be strong at this very local level while non-existent or weak at the municipal level. Bolster et al. [18], investigated the effect of neighbourhood disadvantage on income dynamics using bespoke environments of different scales. They too found that the local level had a stronger association with individual outcomes.

The concept of bespoke environments has been applied in epidemiology at only a limited scale. Frank et al. [19] used this approach to assess the effect of the neighbourhood environment on walking behaviour and obesity. Each household was designated an area of one kilometer around the home. They found that the greater the diversity in land use within the bespoke environment, the lower the risk of obesity. Propper et al. [20] also used bespoke environments in their study on local neighbourhood conditions and mental health. The bespoke environment consisted of the area around each individual that contained the nearest 500–800 people. They found that the prevalence of common mental disorders was related to the socio-economic composition of the surrounding population, although the impact was limited. Maas et al. [21] used bespoke environments to measure the amount of green space in people’s direct residential environment. A weak positive relationship was found with levels of physical activity.

To our knowledge, this study is the first in using bespoke environments to assess the association between socio-economic environment and self-reported health. The main aim of this study is to take into account scale effects and boundary effects when assessing the relationship between socio-economic environment and self-reported health in Amsterdam. The analysis consisted of three steps. First, we compared bespoke environments defined at eight different scales, with a radius ranging from 50 meters to 1500 meters, and assessed whether the association between socio-economic factors and self-reported health was strongest at smaller scales. Next, we distinguished between areas that were socio-economically homogeneous and heterogeneous and assessed whether the association between socio-economic factors and self-reported health was stronger among homogeneous areas. Finally, we compared the results with analysis based on administrative areas and assessed whether the bespoke approach showed a stronger association between socio-economic area and self-reported health.

## Materials and Methods

### Ethics Statement

The interview survey data were obtained and analysed anonymously. As the Dutch Act on Medical Research Involving Human Subjects (WMO) does not apply to this study, an official approval of this study by the Medical Ethics Review Committee was not required.

### Data

The data was obtained from the 2009 “State of the City” survey conducted by the Municipality of Amsterdam’s Department of Research and Statistics. The State of the City surveyed 4351 inhabitants of Amsterdam. Stratified sampling was used to ensure that residents of all districts and ethnic groups within Amsterdam were represented, and respondents from five socially deprived neighbourhoods were oversampled. Data was collected by telephone (29 percent of all respondents), face-to face interviews (16 percent) and postal questionnaires (56 percent), with response rates of 34, 30 and 14 percent respectively (because the documentation of the source data mentions only rounded percentages the sum is not equal to 100). In the analysis we excluded respondents living in buffers with less than 10 inhabitants and/or less than 10 houses, because for these areas we could not obtain valid measures of the socio-economic environment. In the final analysis 4131 respondents were included.

The survey asked respondents about their living situation, such as housing and neighbourhood conditions, socioeconomic position and health. Perceived health status was measured by the response to the question, ‘All in all, would you say your health is excellent, good, fair or poor?’ The answers were classified into two categories: excellent/good and fair/poor. From the same survey, we obtained data on characteristics of the respondents that were used as control variables at the individual level. These include age, sex, ethnicity, household composition, educational level, income level, receipt of social benefits, home ownership and a measure of general wealth (whether the respondent experienced difficulties living on his or her current household income).

To measure the socio-economic characteristics of each respondent’s environment, we used integral socio-economic registries maintained by the Municipality of Amsterdam. The registries were obtained by aggregating information from individual residents, households or houses to the level of 6-digit postcodes. A 6-digit postcode area, originally used for postal delivery, is the smallest geographical unit available. In urban areas, these units are sized approximately 50×50 meters and include 10 to 20 households. For each postcode area, we constructed three socio-economic variables: the percentage of residents receiving a social benefit (unemployment or welfare), the percentage of social housing, and the average property value of houses.

**Figure 1:**
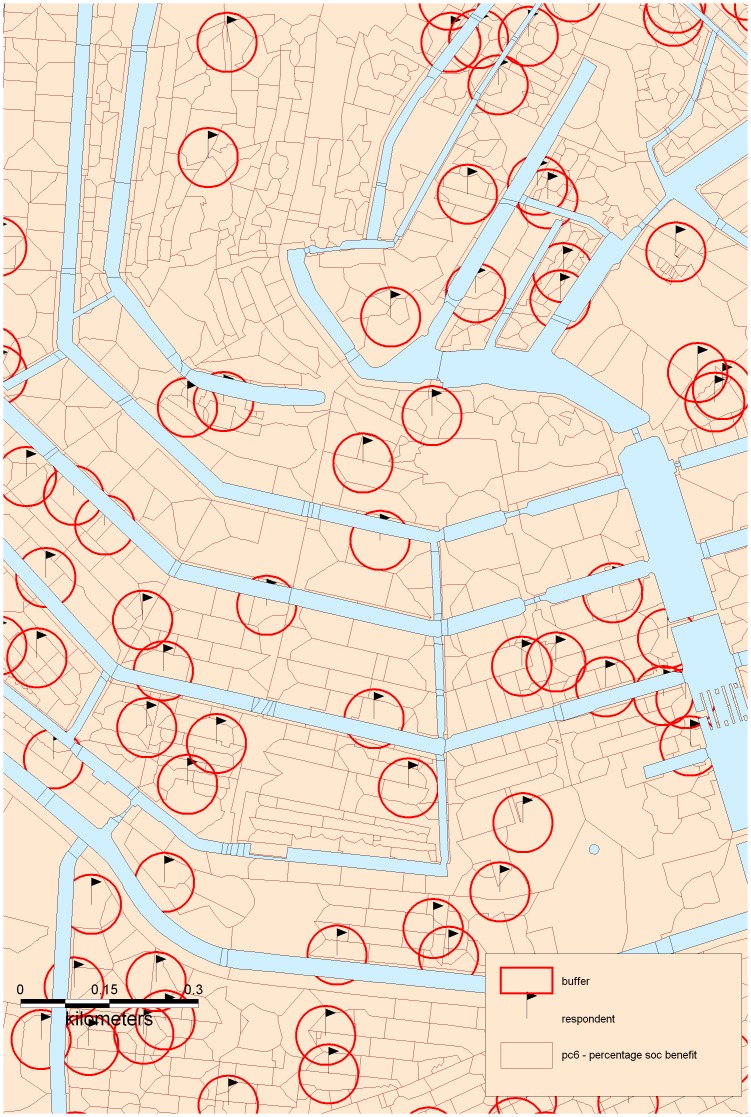
Data aggregation from 6-digit postcode areas to 50 meters radius bespoke environments.

We constructed ‘bespoke environments’ or ‘buffers’ for each respondent using Geographic Information Systems (GIS) based on the central point location of the respondent’s six-digit postal code. Buffers of eight sizes, with a radius ranging from 50 to 1500 meters, were created around each respondent. Finally, the socioeconomic characteristics of each of these buffers were estimated based on the data aggregated by postcode. Postcode areas that were only partially located within the buffer were weighted based on the percentage of the area contained. For this process, we performed an overlay operation, which joins data layers based on common geographical location. This approach is illustrated in figure 1.

In addition, we optimized the geographic delimitation of the buffers, and the measurement of their socioeconomic characteristics, by taking into account natural barriers. These barriers included the Amstel River, the IJ River and the Ring Road (highway). The resulting, more strictly delimited areas were expected to correspond more closely to the mental map and the immediate living environment of the respondents.

For further analyses, we classified the buffers based on whether they were socioeconomically homogeneous or heterogeneous. This determination was made by calculating the standard deviation of each of the socioeconomic variables for the postcode areas within the buffer. The buffer was considered relatively homogeneous if the standard deviation was smaller than average for at least two socioeconomic variables. All other buffers were considered to be heterogeneous.

We also analysed respondents’ administratively defined areas for comparison with the bespoke environment. We used three types of administrative areas: the 4-digit postcode area (on average 2.5 km^2^, or approximately 1.6 km by 1.6 km); districts (referred to as ‘wijken’ in the Netherlands, on average 1.8 km^2^); and wards (‘buurten’, on average 0.4 km^2^). Wards and districts in Amsterdam are considered to be socioeconomically homogeneous. The boundaries for wards are primarily determined by physical boundaries and often correspond to specific periods of construction. Wards are a common unit of geographical analysis by statistical bureaus and municipal offices.

Table 1 shows the extent of geographical variation in the three socioeconomic variables based on the spatial unit (bespoke and administrative). If the size of the buffer increases, the standard deviation of socioeconomic variables decreases. The standard deviations for the percentage of the population receiving a social benefit decreased from 9.1 to about 3.6; the standard deviation for average property value decreased from 9.5 to 5.4. The standard deviations for the percentage of the population living in social housing are high at a small buffer size, but quickly decrease with increasing buffer size.

**Table 1:** Mean and variation of contextual variables.

Spatial unit	Area (km^2^)	Percentage receiving social benefit		Percentage of social housing		Average property value	
		mean	standard deviation	mean	standard deviation	mean	standard deviation
Buffer 50	0.0078	15.37	9.05	56.55	37.28	23.35	9.47
Buffer 100	0.0311	15.49	7.81	56.70	33.52	23.96	9.05
Buffer 150	0.0699	15.49	7.15	56.82	30.59	24.51	8.75
Buffer 200	0.1244	15.53	6.71	56.94	28.24	24.99	8.53
Buffer 300	0.2798	15.61	6.08	57.04	24.92	25.77	8.22
Buffer 600	1.1191	15.52	5.09	56.85	20.45	27.66	7.39
Buffer 1000	3.1087	15.27	4.25	56.14	17.34	29.28	6.43
Buffer 1500	6.9945	14.77	3.57	54.48	15.42	30.35	5.43
Ward	0.4826	15.45	6.41	56.36	26.83	25.58	8.66
District	1.8573	15.58	5.53	56.89	23.11	26.89	7.92
Postcode 4	2.5104	15.25	5.12	56.34	20.99	29.40	8.03

### Statistical Analysis

The relationship between socio-economic characteristics of areas and self-reported health was assessed using logistic regression analysis, with fair/poor health as the dependent variable. We controlled for age, sex, ethnicity and household composition (model 1), as well as for education, income, receipt of social benefit, home ownership and the proxy for wealth (model 2). The results of these models are expressed in terms of odds ratios, which are derived from the regression coefficient for the socioeconomic characteristics. The 95 percent confidence intervals are derived from the standard errors of the regression coefficients.

To enable comparison between the different buffer sizes, we also present the odds ratios corresponding to standardized regression coefficients (which is equivalent to transforming the socioeconomic variables into z-scores before performing a logistic regression). These odds ratios can be interpreted as the increase in the odds of fair/poor perceived health if the socioeconomic level of a neighbourhood changes with one standard deviation. This measure takes into account the large differences in standard deviation according to buffer size (table 1).

In order to quantify the explanatory power of socioeconomic characteristics of areas, we also applied a regression strategy involving two steps: first we included only the individual-level characteristics, and next we added the socioeconomic characteristics of areas. Using Nagelkerke R^2^ and AIC, we quantified the increase in explained variance by adding the latter terms.

## Results

Table 2 illustrates the percentage of respondents reporting fair/poor health, broken out by the respondents’ individual characteristics. Fair/poor health is more often reported by single parent families (33.9 percent), non-western migrants (on average 33.5), respondents with no education (55) or a low educational-level (33.5), lower income groups (about 50), those receiving social benefit (62.9) and those having difficulties in making ends meet (61.2).

**Table 2:** Number of respondents and percentage reporting fair/poor health by individual characteristics.

Individual variable	N	% reporting poor health
**Sex**		
Male	1821	23.7
Female	2310	25.9
**Age**	4131	
**Household composition**		
Single-parent family	322	33.9
Two adults with child	1244	21.8
Two adults without child	1210	22.5
Single	1172	28.7
Other	183	23.0
**Ethnicity**		
Natives	2209	18.9
Surinamese	308	35.1
Atillean	63	31.7
Turks	343	38.5
Moroccans	461	33.6
Other non-western immigrants	192	30.2
Western immigrants	458	22.1
Rest of Asia	97	39.2
**Education**		
No education	496	55.0
Low	791	33.5
Medium	971	20.9
High	1578	10.6
Other	295	41.4
**Income net (Euros)**		
700	192	44.8
700–1000	442	50.0
1000–1350	503	38.2
1350–2050	831	20.6
2050–3200	757	14.8
3200 and more	590	6.3
Missing	816	25.9
**Receiving social benefit**		
Yes	267	62.9
No	3864	22.3
**Home ownership**		
No owner	2558	29.7
Owner	1518	16.7
**Living on household income**		
Very difficult	209	61.2
Quite difficult	660	44.8
Difficult	536	33.0
Quite easy	784	20.0
Easy	1327	14.7
Very easy	467	8.8
Missing	148	34.5

Table 3 quantifies the explanatory power of models including socioeconomic characteristics of areas, in terms of increase in percentage of variance explained and decrease of AIC. The explanatory power is strongest for small buffers, and it declines with increasing buffer size. For the percentage of residents living on social benefit, the percentage explained declines from 1.3 percent for 50 meter buffer size to 0.3 percent for 1500 meter buffer size. Notably, the percentage explained when the three socioeconomic variables are combined hardly exceeds the percentage that could already be explained by variable on residents living on social benefit.

**Table 3:** Changes in R^2^ and AIC by inclusion of neighbourhood-SES variables compared to a model without neighbourhood-SES*.

Buffer size	Percentage receiving social benefit		Percentage of social housing		Average property value		Together	
	R^2^ (%)	Increase	R^2^	Increase	R^2^	Increase	R^2^	Increase
50	18.5	1.3	18.4	1.2	18.0	0.8	18.8	1.6
100	18.3	1.1	18.2	1	17.8	0.6	18.5	1.3
150	18.1	0.9	18.1	0.9	17.7	0.5	18.2	1
200	18.1	0.9	18.0	0.8	17.6	0.4	18.2	1
300	17.9	0.7	17.8	0.6	17.6	0.4	17.9	0.7
600	17.7	0.5	17.7	0.5	17.5	0.3	17.8	0.6
1000	17.6	0.4	17.5	0.3	17.3	0.1	17.6	0.4
1500	17.5	0.3	17.5	0.3	17.3	0.1	17.5	0.3
	**AIC**	**Decrease**	**AIC**	**Decrease**	**AIC**	**Decrease**	**AIC**	**Decrease**
50	4120	38	4122	36	4134	24	4114	44
100	4124	34	4128	30	4140	18	4124	34
150	4130	28	4133	25	4143	15	4131	27
200	4131	27	4135	23	4146	12	4134	24
300	4139	19	4142	16	4148	10	4141	17
600	4143	15	4146	12	4152	6	4146	12
1000	4148	10	4150	8	4156	2	4152	6
1500	4151	7	4152	6	4158	0	4154	4

* base model without neighbourhood-ses (age, sex, household composition, ethnicity: Nagelkerke R^2^ = .172; AIC = 4158).

Table 4 presents the effect of controlling for individual-level socioeconomic variables. We pay particular attention to the standardized odds ratio of columns 4 and 5. For example, the odds ratio in column 4 is 1.30 for the share of people living on social benefit within 50-meter buffers. This implies that if the share of people living on social benefit increases by 1 standard deviation (in this case 9 percent; see table 1), the odds of fair/poor health increases by 30 percent. After controlling for individual-level socioeconomic variables, this odds ratio declines to 1.10. Generally, after controlling for all individual-level variables, the association between health and socioeconomic factors is strongest at small buffer sizes. Statistically significant associations are found only for 50-meter buffers and 100-meter buffers (for the percentage of social housing). For buffer sizes larger than about 200 meters, the associations are not statistically significant. Moreover, above 200 meters, the odds ratios in columns 3 and 5 do not provide indications of a consistent relationship with buffer size.

**Table 4:** Comparison of the effects of the contextual variables between model 1 (base model) and model 2 (extensive model) at different scales.

Buffer size	Odds ratio’s (95% CI)		Standardized OR	
	Model 1	Model 2	Model 1	Model 2
receiving social benefit (%)				
50	1.029 (1.018;1.039)	1.011 (1.001;1.022)	1.30	1.10
100	1.032 (1.022;1.043)	1.009 (0.997;1.021)	1.28	1.07
150	1.032 (1.020;1.045)	1.007 (0.993;1.021)	1.25	1.05
200	1.033 (1.021;1.046)	1.007 (0.993;1.021)	1.24	1.05
300	1.032 (1.017;1.046)	1.005 (0.989;1.021)	1.21	1.03
600	1.033 (1.017;1.050)	1.007 (0.989;1.023)	1.18	1.04
1000	1.032 (1.012;1.053)	1.008 (0.988;1.028)	1.14	1.03
1500	1.034 (1.011;1.056)	1.014 (0.991;1.038)	1.13	1.05
social housing (%)				
50	1.007 (1.005;1.009)	1.002 (1.000;1.004)	1.30	1.08
100	1.007 (1.005;1.009)	1.002 (1.000;1.004)	1.26	1.07
150	1.007 (1.005;1.009)	1.001 (0.997;1.005)	1.24	1.03
200	1.007 (1.005;1.009)	1.001 (0.997;1.005)	1.22	1.03
300	1.007 (1.003;1.011)	1.001 (0.997;1.005)	1.19	1.03
600	1.007 (1.003;1.011)	1.001 (0.997;1.005)	1.15	1.02
1000	1.007 (1.003;1.011)	1.001 (0.995;1.007)	1.13	1.02
1500	1.007 (1.001;1.013)	1.001 (0.995;1.007)	1.11	1.02
average property value (in 10.000 Euros)*				
50	1.023 (1.013;1.033)	1.006 (0.996;1.016)	1.25	1.05
100	1.020 (1.011;1.031)	1.004 (0.994;1.014)	1.20	1.04
150	1.019 (1.007;1.030)	1.003 (0.993;1.013)	1.18	1.03
200	1.017 (1.008;1.029)	1.002 (0.992;1.012)	1.16	1.02
300	1.017 (1.007;1.028)	1.003 (0.991;1.015)	1.15	1.02
600	1.015 (1.005;1.026)	1.006 (0.994;1.018)	1.12	1.04
1000	1.010 (0.999;1.022)	1.006 (0.992;1.020)	1.06	1.04
1500	1.009 (0.995;1.024)	1.006 (0.990;1.021)	1.05	1.03

* For average property value, the OR is inverted to make it more directly comparable to the other SES indicators. The OR represents the increase in odds of poor health if property value decreases with 10,000 Euro’s.

In table 5, the results are compared across buffers that are relatively homogeneous in terms of the percentage of people receiving social benefits. In this sub-set of buffers, the association with health is stronger. Standardised odds ratios are highest for homogeneous buffers of 300-meters or less, up to an odds ratio of 1.15 for homogeneous buffers of 50-meters. No associations were observed in the larger buffers, irrespective of their degree of homogeneity. For the other two socioeconomic variables (percentage of social housing; property values), we also found that associations were evident only in relatively homogeneous buffers of 300 meters and smaller (results not shown).

**Table 5:** Comparison of neighbourhood effects between relatively homogeneous buffers and all buffers together.

Percentage of people receiving social benefit					
Buffer size	Group	Odds ratio’s (95% CI)		Standardized OR	
		Model 1	Model 2	Model 1	Model 2
50	homogeneous	1.035 (1.021;1.050)	1.018 (1.004;1.032)	1.37	1.15
	all	1.029 (1.018;1.039)	1.011 (1.001;1.002)	1.30	1.10
100	homogeneous	1.038 (1.021;1.054)	1.017 (0.999;1.035)	1.34	1.12
	all	1.032 (1.022;1.043)	1.009 (0.997;1.021)	1.28	1.07
150	homogeneous	1.027 (1.008;1.045)	1.009 (0.989;1.029)	1.21	1.06
	all	1.032 (1.020;1.045)	1.007 (0.993;1.021)	1.25	1.05
300	homogeneous	1.019 (0.999;1.040)	0.998 (0.978;1.020)	1.12	0.99
	all	1.032 (1.017;1.046)	1.005 (0.989;1.021)	1.21	1.03
600	homogeneous	1.026 (1.011;1.063)	1.016 (0.989;1.044)	1.13	1.08
	all	1.033 (1.017;1.050)	1.007 (0.989;1.023)	1.18	1.04

Table 6 explores whether analysis using administratively defined areas yields different results compared to analysis using bespoke environments. The results turn out to be similar: when socioeconomic factors are measured at the level of the smallest administrative unit, the ward, they can explain most of the variance in fair/poor health. The percentage explained at the ward level is about as large as when socioeconomic factors are measured at the level of buffers of 200 meters or smaller (cf. table 3). The AIC results indicate the same: the model improves if neighbourhood-SES variables are included and the effect decreases as administrative scales increase.

**Table 6:** Changes in R^2^ and AIC for three neighbourhood-SES variables at different administrative scales compared to a model without neighbourhood-SES*.

	% social benefit		% social housing		Average property value		Together	
administrative-zone	R^2^ (%)	Increase	R^2^	Increase	R^2^	Increase	R^2^	Increase
ward (0.4 km^2^)	17.9	0.7	17.9	0.7	17.7	0.5	18.1	0.9
combination (1.8 km^2^)	17.5	0.3	17.4	0.2	17.5	0.3	17.7	0.5
postcode 4 (2.5 km^2^)	17.5	0.3	17.5	0.3	17.4	0.2	17.6	0.4
	**AIC**	**Decrease**	**AIC**	**Decrease**	**AIC**	**Decrease**	**AIC**	**Decrease**
ward (0.4 km^2^)	4135	23	4137	21	4143	15	4131	27
combination (1.8 km^2^)	4137	21	4138	20	4152	6	4132	25
**postcode 4 (2.5 km^2^)**	4150	8	4151	7	4154	4	4148	10

* base model without neighbourhood-ses (age, sex, household composition, ethnicity: Nagelkerke R^2^ = .172; AIC = 4158).

**Figure 2:**
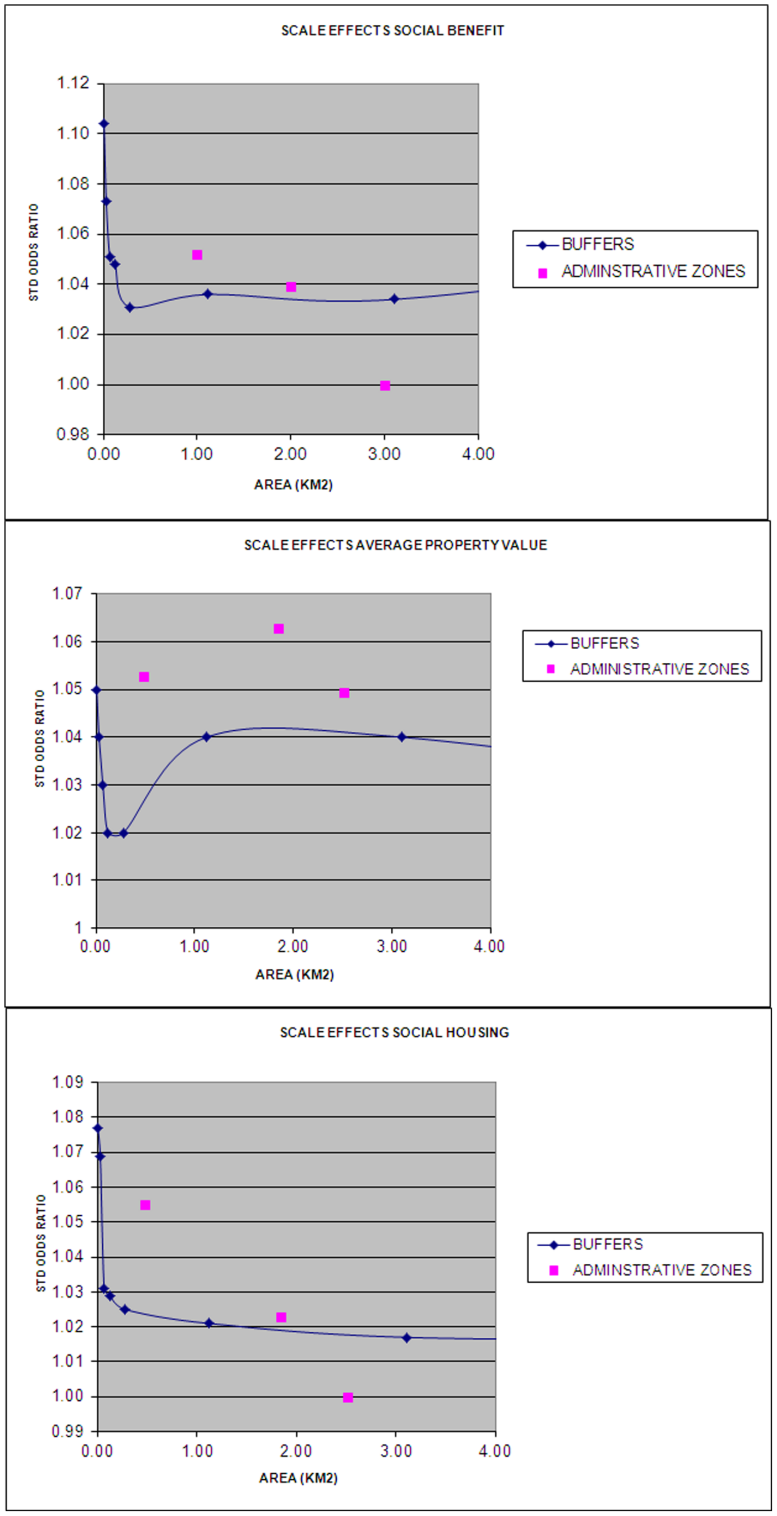
The strength of socioeconomic area effects according to scale.

Figure 2 shows the standardised odds ratios, as estimated for different buffers. The odds ratios are plotted against the average size of the surface of the buffers. In general, the odds ratios decrease with increasing area surface of buffers. This implies that the association between health and socioeconomic factors is weaker when the latter are measured to larger buffers. For average property value, this trend is less consistent as odds ratios sharply increase for buffers smaller than 600 meter buffers. For the other two area characteristics, the association becomes consistently weaker with increasing area surface.

In addition, in Figure 2, a comparison can be made between buffers and administratively defined areas, while taking area surface into account. Analyses at the 4 digit postcode yield smaller effect estimates as compared to analyses using buffers of about similar size. However, when socioeconomic factors are measured at the level of districts, they perform equally well as socioeconomic variables measured at the level of buffers of a comparable size.

## Discussion

Previous studies may have underestimated the association between health and socioeconomic characteristics of areas due to scale and boundary effects. We aimed to address these effects by using “bespoke environments” or “buffers” to study the relationship between health and the surrounding socio-economic environment. By comparing buffers of different sizes, we observed that the association between socio-economic environment and self-reported health could be demonstrated only for small buffers with a radius of 50 or 100 meters. Stronger associations were observed in analyses that only compared relatively homogeneous areas. When socioeconomic factors were measured to small administrative units (wards), they performed equally well as socioeconomic variables measured at the level of buffers of comparable size.

### Evaluation of Methodology

Our method and results should be considered in the light of the modifiable areal unit problem (MAUP). The MAUP states that area-level effects are dependent on the form, size and location of the sub-areas used. This dependency is particularly important when using administratively defined areas. Administrative zones have a form, size and location that are often quite arbitrary. In studies using administrative areas, the results therefore could be strongly sensitive to the precise delineation of these areas [22].

Theoretically, bespoke environments should solve some aspects of this problem. By using bespoke environments, all areas have the same form (distances are equal in all directions) and location (each area is based around the center point of individual respondents), thus avoiding potential boundary effects. In addition, the size aspect can be addressed by using bespoke environments of different sizes.

The use of bespoke environments as a geographic method might however bring new challenges as well. Because buffers overlap, especially the larger ones, observations for individual respondents are not entirely independent. Failure to take into account this dependency may result in overestimation of the precision and statistical significance of the area-effects. The use of multi-level models, using a restricted number of environments, would address this problem. However, when applying bespoke environments, such models cannot be easily integrated as respondents do not share identical environments and thus cannot be aggregated into the same high-order level category. We would like to note that, in our analyses, the strongest effects were observed at a smaller scale (50 meters) where buffers rarely overlapped.

We might have failed to control for potentially important confounders at the area level, such as land use mix, or noise nuisance caused by Schiphol Airport. We checked for area-level confounding by mapping the residuals of the regression analyses, with full control for individual-level variables. However, we did not observe spatial clusters of residuals, suggesting that there are no area-level confounders that could have biased our results to a significant extent.

In the analysis, we aimed to control for individual-level demographic and socioeconomic characteristics that could be considered to be potential confounders to the association between health and the surrounding socioeconomic environment. As controlling for these characteristics had an important effect on our effect estimates, we cannot exclude the possibility that more detailed control would remove even more of the area-level effect. At the same time, we would like to stress that we already had controlled both for education, income and wealth (by proxy), and that the potential for residual confounding by SES thus seems limited. However, we cannot exclude potential confounding by other factors and capabilities that may determine where people can choose to live [23].

The overall response rate to the survey was only 23 percent. It is documented that, in general, non responders are often young, of non-Western origin and have a low income [24]. These characteristics were strongly related to self perception of health. Given these relationships, we cannot exclude the possibility that selective non-response may have biased our estimates of the association between health and the socioeconomic environment. Most likely, we think that this association may have been underestimated to some extent.

Studies comparing administrative areas and alternative definitions of a neighbourhood found similar associations with health outcomes irrespective of the way in which the neighbourhood boundaries were defined [25], [26]. This corresponds to our finding that the analysis of wards yielded similar results as the analysis with similarly-sized buffers. However, we might have expected associations to be stronger with the buffers, as buffers may be a better representation of one’s immediate living environment and activity space. Our results however suggest that administrative areas that are defined with regards to socioeconomic and geographic criteria, such as wards (in the case of Amsterdam), may function equally well.

By using GIS techniques we had the opportunity to construct residential areas on a very local scale. We observed this to be an important advantage, as the association between socioeconomic variables and health was found to be the strongest, and only demonstrable with statistical significance, at the level of very small buffers (50- or 100-meter). In addition, GIS techniques make it possible to perform additional geographic operations such as measuring the degree of homogeneity of areas. This offered the opportunity to restrict the analysis to a subset of areas with greater contrast in socioeconomic conditions.

### Interpretation and Comparison to Previous Studies

Other studies have also observed that the association between health and area-level socioeconomic characteristics was stronger in smaller areas. For example, one Dutch study assessed the effects of area-level socio-economic factors on mortality within postcode areas, districts, and wards. That study showed that differences in mortality chance of men were most pronounced at the lowest scale level of postcode areas [27].

We observed that the effect of area-level socioeconomic factors was small in comparison to the effects of individual-level socioeconomic variables on health (cf. table 2 and 4). A relatively small effect was also found in other Dutch studies [4], [28] and should possibly be considered in a national context. We postulate that effects of socio-economic conditions of areas may be small in a welfare state such as the Netherlands due to, among other factors, social housing policies and urban renewal schemes that that have limited sharp differences in living conditions amongst its population.

The fact that effects are observed only at the level of small (50–100 meter) buffers is suggestive of an effect of factors with a highly local reach. Among these, social networks might play an important role. In the case of voting behaviour, Johnston [17] and McAllistar [15] found clear links between local milieus and how people behave. Those who live in relative close proximity are more likely to think and act in similar ways. Other localized factors may include neighbourhood-level psycho-social stressors (e.g., nuisance from neighbours, feeling unsafe, drug abuse, etc.), many of which have been found to be related to self-rated health, including in Amsterdam [29]. Generally, these stressors may produce health effects on local scales, especially in socio-economically deprived areas [30].

### Conclusions

To conclude, this study observed scale effects to be highly important when studying socio-economic area effects on health. The measurement of socioeconomic factors for large areas might result in a substantial underestimation, or even a negligence, of the effects of socioeconomic environment on health. The results stress the importance of using micro-scale data on the environment as well as health outcomes in order to study the relationship between these two. When such data are available, the methodology of bespoke environments could be applied to many environmental features and health-related outcomes. An important advantage of this methodology is that the buffer width can be tuned to the scale at which processes are expected to operate – whether a few meters or a few kilometres. The most relevant scale is likely to vary based on the health outcome and population group (e.g. children vs. middle-aged men) being measured. Through scaling, bespoke environments add a new dimension to study environment and health.
